# Spontaneous Reports of Serious Adverse Drug Reactions Resulting From Drug–Drug Interactions: An Analysis From the French Pharmacovigilance Database

**DOI:** 10.3389/fphar.2020.624562

**Published:** 2021-03-24

**Authors:** Louis Létinier, Amandine Ferreira, Alexandre Marceron, Marina Babin, Joëlle Micallef, Ghada Miremont-Salamé, Antoine Pariente, on behalf of the French Network of Pharmacovigilance Centres

**Affiliations:** ^1^ Univ. Bordeaux, INSERM, BPH, U1219, Team Pharmacoepidemiology, Bordeaux, France; ^2^ CHU de Bordeaux, Pole de Santé Publique, Service de Pharmacologie Médicale, Centre de Pharmacovigilance de Bordeaux, Bordeaux, France; ^3^ Service de Pharmacologie Toxicologie et CRPV, CHU, Angers, France; ^4^ CRPV Marseille Provence Corse, Service Hospitalo-Universitaire de Pharmacologie Clinique et Pharmacovigilance, Assistance Publique Hôpitaux de Marseille, Marseille, France; ^5^ Aix Marseille Université, Institut des Neurosciences des Systèmes, INSERM 1106, Marseille, France

**Keywords:** drug–drug interaction, pharmacovigilance, spontaneous reporting, adverse drug reaction, hemorrhages, antithrombotic agents

## Abstract

Few data are available on the clinical impact of drug–drug interactions (DDIs). Most of the studies are limited to the analysis of exposure to potential DDI or the targeted impact of the combination of a few drugs or therapeutic classes. The analysis of adverse drug reaction (ADR) reports could be a mean to study generally the adverse effects identified due to a DDI. Our objective was to describe the characteristics of ADRs resulting from DDIs reported to the French Pharmacovigilance system and to identify the drugs most often implicated in these ADRs. Considering all ADR reports from January 01, 2012, to December 31, 2016, we identified all cases of ADR resulting from a DDI (DDI-ADRs). We then described these in terms of patients’ characteristics, ADR seriousness, drugs involved (two or more per case), and ADR type. Of the 4,027 reports relating to DDI-ADRs, 3,303 were related to serious ADRs. Patients with serious DDI-ADRs had a median age of 76 years (interquartile range: 63–84); 53% were male. Of all serious DDI-ADRs, 11% were life-threatening and 8% fatal. In 36% of cases, the DDI causing the ADR involved at least three drugs. Overall, 8,424 different drugs were mentioned in the 3,303 serious DDI-ADRs considered. Altogether, drugs from the “antithrombotic agents” subgroup were incriminated in 34% of serious DDI-ADRs. Antidepressants were the second most represented therapeutic/pharmacological subgroup (5% of serious DDI-ADRs). Among the 3,843 ADR types reported in the 3,303 serious DDI-ADRs considered, the most frequently represented were hemorrhage (40% clinical hemorrhage; 6% biological hemorrhage), renal failure (8%), pharmacokinetic alteration (5%), and cardiac arrhythmias (4%). Hemorrhagic accidents are still an important part of serious ADRs resulting from DDIs reported in France. The other clinical consequences of DDIs seem less well identified by pharmacovigilance. Moreover, more than one-third of serious DDI-ADRs involved at least three drugs.

## Introduction

Exposure to drug–drug interactions (DDIs) is a well-known public health issue (Becker et al., 2008; Létinier et al., 2019). Most of the studies conducted on this subject show an increase in the frequency of potential and clinically relevant DDI over the years, associated with the aging of the population and polymedication (Nobili et al., 2009; Maher et al., 2014; Guthrie et al., 2015). Some pharmacoepidemiological studies have tried to estimate the clinical impact of these DDIs, but it remains difficult given the multiplicity of the drugs involved and the diversity of potential adverse events (Magro et al., 2012; Hennessy et al., 2016). Pharmacovigilance databases of spontaneous reporting can provide complementary information to investigate this impact, as they can allow describing the adverse drug reactions (ADRs) as DDI induced. Despite this, most of the recent publications in the field have not focused on such description but instead on the detection of safety signals regarding unknown DDIs (Jiang et al., 2015; Vilar et al., 2017); in terms of general prescription, we could identify only one study performed in a French region that has shown the reported ADR related to DDI mainly concerning renal failure and hemorrhages (Duval, 2017). The other existing publications were mainly investigating specifically some targeted drugs (Montastruc et al., 2012; Suzuki et al., 2015; Schlit et al., 2017), and none was identified that considered more than two drugs when exploring the consequences of DDIs in terms of ADRs, although this could frequently occur in patients.

In this context, our objective was to describe the characteristics of adverse drug reactions resulting from drug–drug interactions (DDI-ADRs) reported to the French network of Pharmacovigilance Centers and to identify the drugs most often implicated in these. We additionally aimed at describing the DDIs involving more than two drugs.

## Materials and Methods

### Dataset

The French Pharmacovigilance system is based on a network of 31 regional centers. In those, ADRs reported by health professionals or patients are evaluated by trained pharmacologists before they are entered into the French Pharmacovigilance Database (BNPV). The ADR declaration is mandatory for physicians and pharmacists but voluntary for patients. In practice, it is especially recommended to report unexpected or serious ADRs. There are different supports and forms of declarations, including an increasingly used national web portal. Each year, around 40,000 ADR reports are collected by this system. The assessment includes a determination of whether ADR is serious and whether it results from a DDI. In this study, we considered all cases of DDI directly coded as such and all cases of ADR, pregnancy exposure, drug misuse or abuse, weaning, or overdoses with at least one drug identified as having caused an interaction. To be serious, an ADR needs to result in death, life-threatening condition, hospitalization (or prolongation of existing hospitalization), persistent or significant disability or incapacity, congenital abnormalities/birth defect, or another significant medical event. If the ADR results from a DDI, the pharmacologist will determine which drugs are suspected of being related to the ADR. Moreover, it may be that more than two drugs are considered suspect, defined here as multiple DDI.

### Population and Cases

Considering all ADR reports entered in the BNPV from January 01, 2012, to December 31, 2016, we identified all cases of serious and nonserious DDI-ADRs. We described general characteristics of the ADR reports considered as DDI and of other ADRs. We then described serious DDI-ADRs in terms of patients’ characteristics, ADR seriousness type, drugs involved (two or more per case), and ADR type. Drugs involved were described according to the Anatomical Therapeutic Chemical classification (ATC) Third level (therapeutic/pharmacological subgroups) and Fifth level (chemical substance). In the BNPV, ADRs are coded using the Medical Dictionary for Regulatory Activities (MedDRA) Preferred Terms (PTs). ADR type was consequently studied using Standardized MedDRA Queries (SMQs) or *ad hoc* defined sets of MedDRA terms when no SMQ was available.

### Statistical Analysis

The general characteristics of patients for whom a DDI-ADR was reported were described, as were those of all patients for whom an ADR was reported, whatever the mechanism. For DDI-ADRs, a description was also performed according to the ADR seriousness (serious *vs.* nonserious DDI-ADRs). Finally, the description of serious DDI-ADRs was later stratified according to the type of drugs and to the type of ADR.

In addition, a detailed description and visualization of the drug therapeutic/pharmacological subgroups involved in the most frequent DDI-ADR were performed.

Quantitative variables were described in terms of the median and interquartile range (IQR), and qualitative variables were described as proportion, including missing data. Due to the very observational nature of the data and to the expected existence of differences between groups, we did not perform statistical testing and only performed comparative description when studying different groups of patients or serious vs. nonserious DDI-ADRs.

All analysis and visualization were performed using RStudio Version 1.0.143—^©^ 2009–2016 RStudio, Inc.

## Results

### General Description of ADR Reports

From January 01, 2012, to December 31, 2016, 190,261 ADRs were reported to the BNPV, of which 4,027 were identified as resulting from a DDI (2.1%) (Table 1). Then, 3,303 of these were identified as serious DDI (82%) and 274 were fatal (7%). These proportions of severity are higher for DDIs than for all 190,261 ADRs reported to the BNPV during the study period (63% of serious cases and 3% of deaths). The median age of patients for whom a DDI-ADR was reported was of 75 years (IQR: 61–84); 48% were women. In the overall population of patients for whom an ADR was reported in the BNPV over the period, the median age was of 57 years (IQR: 42–75); the proportion of women was 55%.

**TABLE 1:** General characteristics of the ADR reports considered as DDI and of the ADR reports not relating to identified DDIs entered in the French Pharmacovigilance Database during the 2012–2016 period.

Characteristics	DDI-ADRs, *n* = 4,027^a^	Not DDI-ADRs *n* = 186,594	all ADRs, *n* = 190,621^a^
	*n* (%)	*n* (%)	*n* (%)
Age in years, median (IQR)	75 (61–84)	56 (41–74)	57 (42–75)
Women	1,948 (48)	102,660 (55)	104,608 (55)
ADR type coding^b^			
DDI-ADR	3,409 (85)	0	3,409 (2)
ADR	487 (12)	171,513 (92)	172,000 (90)
Others	131 (3)	14,721 (8)	14,852 (8)
Seriousness	3,303 (82)	115,711 (62)	119,014 (63)
incl. Death	274 (7)	6,014 (3)	6,288 (3)

^a^ Missing data: DDI-ADRs: age 3.3%; sex 0.2%; reporter type 0.05%; all ADRs: 6,759 for age; 773 for sex, 287 for reporter.

^b^ In the BNPV, ADR can be coded as “ADR” without specification, "DDI", or “other ADR”, which relates to pregnancy exposure, drug misuse or abuse, weaning, or overdoses. The information of DDI can be either straightly coded in this ADR type, in the information and coding relating to drugs involved, that can be tagged as “interacting”.

Among patients for whom a DDI-ADR was reported, those being reported for a serious DDI-ADR appeared older than patients reported for a nonserious one (median age: 76 *vs.* 67) and to be more likely to be more men (53% *vs.* 46%) (Table 2). Among serious DDI-ADRs reports, DDIs involving more than two drugs appeared more frequent (36% *vs.* 29%).

**TABLE 2:** General characteristics of serious and nonserious DDI-ADRs.

Characteristics	Serious, *n* (%) *n* = 3,303	Nonserious, *n* (%) *n* = 724
Age, year, median (interquartile range)	76 (63–84)	67 (50–81)
Female	1,558 (47)	390 (54)
Reporter type		
Specialist physician	2,400 (73)	350 (48)
Generalist practitioner	122 (4)	84 (12)
Pharmacist	735 (22)	226 (31)
Nurse	6 (0)	4 (1)
Lawyer	1 (0)	1 (0)
Other healthcare professionals	13 (0)	5 (1)
Patient	26 (1)	52 (7)
Multiple DDI^a^	1,178 (36)	210 (29)

^a^ More than two drugs identified as being involved in the interaction.

Of all serious DDI-ADRs, 11% were life-threatening and 8% fatal (Table 3). A large part of these cases resulted in or prolonged hospitalization (68%).

**TABLE 3:** Seriousness criteria of serious DDI-ADR *n* = 3,303.

Seriousness Criteria	*n* (%)
Death	274 (8)
Life-threatening	364 (11)
Incapacity	35 (1)
Hospitalization	2,240 (68)
Other significant medical events	390 (12)

### Drugs Involved in Serious Drug–Drug Interactions-Adverse Drug Reactions

Overall, 8,424 different drugs were mentioned as “suspect” or “interacting” in the 3,303 serious DDI-ADRs considered (median: 2.5 drugs per case). Those most frequently incriminated were fluindione (11% of serious DDI-ADRs), aspirin (8%), clopidogrel (3%), warfarin (3%), and amiodarone (3%) (Table 4).

**TABLE 4:** Drugs most frequently found among severe cases, ATC fifth and third level n = 8,484.

Drugs	*n* (%)
ATC fifth level	
Fluindione	895 (11)
Aspirin	690 (8)
Warfarin	244 (3)
Clopidogrel	243 (3)
Amiodarone	224 (3)
Enoxaparin	186 (2)
Amoxicillin^a^	168 (2)
Furosemide	156 (2)
Dabigatran	148 (2)
Escitalopram	125 (1)
ATC third level	
Antithrombotic agents	2,919 (34)
Antidepressants	416 (5)
Antipsychotics	274 (3)
Immunosuppressants	271 (3)
Antiarrhythmics, classes 1 and 3	233 (3)
Opioïds	226 (3)
Antiinflammatory and antirheumatic products, nonsteroids (NSAIDs)	216 (3)
Beta-lactam antibacterials, penicillins	207 (2)
Antiepileptics	165 (2)
High-ceiling diuretics	162 (2)

^a^ With or without clavulanic acid.

Altogether, drugs from the *antithrombotic agents* subgroup were incriminated in 34% of serious DDI-ADRs. *Antidepressants* were the second most represented therapeutic/pharmacological subgroup (5% of serious DDI-ADRs) (Table 4).

### Clinical Consequences of Drug–Drug Interaction and Types of Adverse Drug Reaction

Across the 3,843 ADR types reported in the 3,303 serious DDI-ADRs considered, the most frequently represented were hemorrhage (40%; plus biological hemorrhage, 6%), renal failure (8%), pharmacokinetic alteration (5%), and cardiac arrhythmias (4%) (Table 5). Hemorrhage accounted for 76% of the deaths associated with DDI (Table 6). We, therefore, found an average of 1.16 ADR types per serious DDI-ADRs. Pharmacovigilance declarations may indeed contain more than one ADR type.

**TABLE 5:** ADR 20 most frequent types among serious cases, *n* = 3,843^a^.

Adverse reaction type	*n* (%)
Hemorrhage	1,529 (40)
Renal failure	324 (8)
Hemorrhage (biological)^b^	224 (6)
Pharmacokinetic interaction^c^	193 (5)
Arrhythmia	144 (4)
Fall	123 (3)
Hematologic disorder	120 (3)
Anticholinergic syndrome	117 (3)
Hyperkaliemia	105 (3)
Central nervous system depression	99 (3)
Digestive system disorder	97 (3)
Neurological disorder	96 (3)
Hydroelectrolytic disorders	96 (3)
Drug ineffective	82 (2)
Seizure	78 (2)
Weaning	56 (1)
Rhabdomyolysis	56 (1)
Hepatitis	43 (1)
Death and cardiac arrest	41 (1)
Neuroleptic malignant syndrome	37 (1)

^a^ Some cases contained ADRs belonging to different types.

^b^ ADRs corresponding to MedDRA SMQ: "Hemorrhage laboratory terms" (a change in INR, PTT, or clotting factors). The list of corresponding PTs can be viewed at: http://bioportal.bioontology.org/ontologies/MEDDRA?p=classes&conceptid=20000040

^c^ Increase or decrease in plasma concentration.

**TABLE 6:** ADR 10 most frequent types among fatal cases, *n* = 274^a^.

Adverse reaction type	*n* (%)
Hemorrhage	209 (76)
Renal failure	21 (8)
Cardiac arrest	20 (7)
Pharmacokinetic interaction^b^	12 (4)
Hematologic disorder	10 (4)
Central nervous system depression	9 (3)
Arrhythmia	7 (3)
Nonhemorrhagic shock	7 (3)
Digestive system disorder	6 (2)
Hyperkaliemia	5 (2)

^a^ Some cases contained ADRs belonging to different types.

^b^ Increase or decrease in plasma concentration.

### Drug–Drug Interaction-Adverse Drug Reaction Resulting in Hemorrhages

**FIGURE 1:**
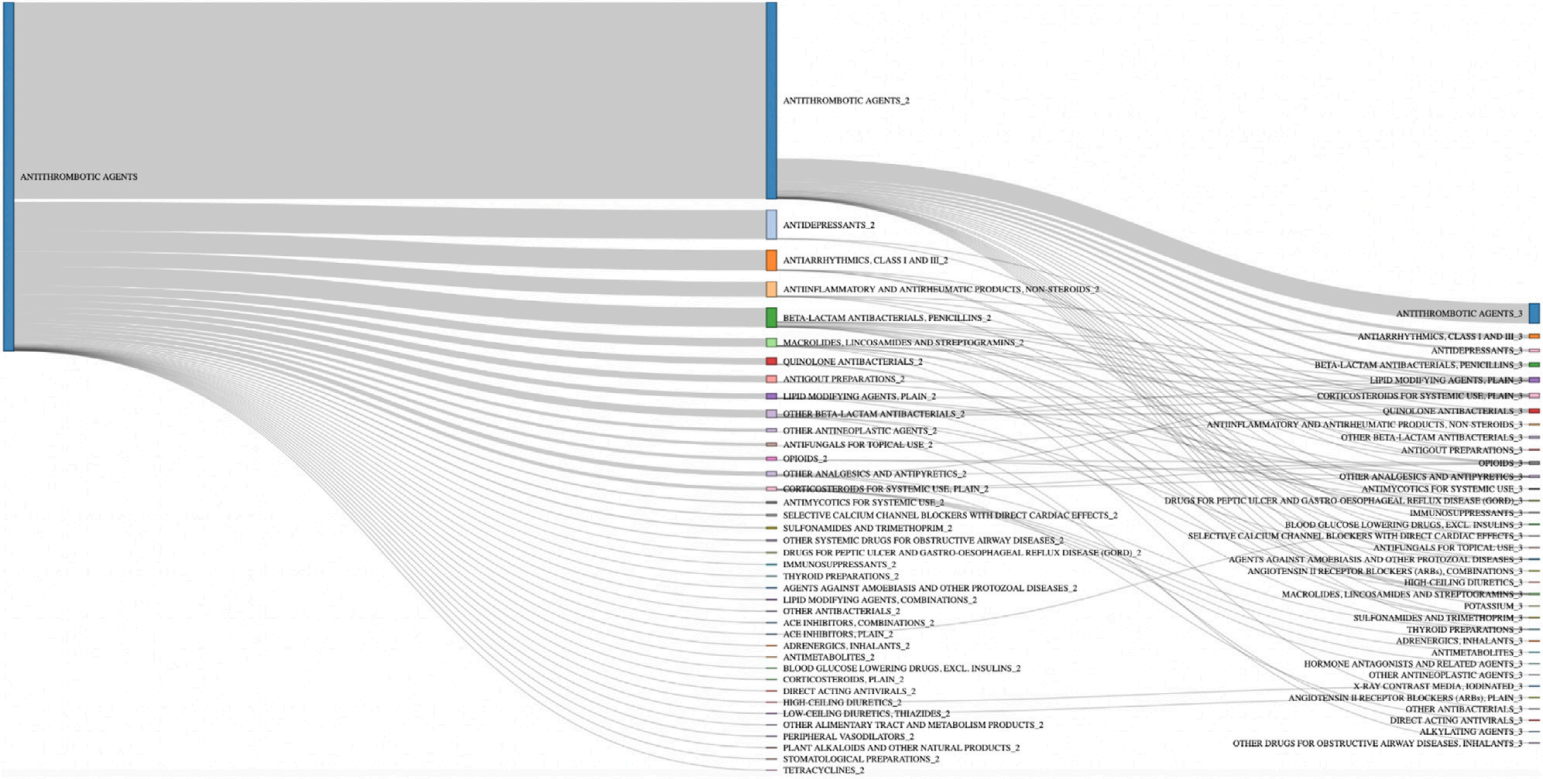
Drug therapeutic/pharmacological subgroup combinations involved in hemorrhage cases with antithrombotic agents *n* = 1,518.

Antithrombotic agents were predominant (70%) among the 3,648 drugs involved in the 1,529 serious DDI-ADRs resulting in hemorrhage. The other main classes represented were antidepressants (5%), antiarrhythmics (4%), and penicillins (3%) (Table 7). Among these cases of hemorrhage, 1,518 (99%) concern at least one antithrombotic agent and the majority of DDIs involved two antithrombotic agents (*n* = 856, 56%) (Figure 1). The other frequently encountered associations concerned antithrombotics + antidepressants (*n* = 126, 8.2%; antithrombotics + escitalopram, *n* = 42 cases, 2.8%), antithrombotics + antiarrhytmics (*n* = 89, 5.8%), antithrombotics + penicillins (n = 84, 5.5%), and antithrombotics + NSAIDs (n = 66, 4.3%). Finally, 254 DDI-ADRs resulting in hemorrhage (17%) involved more than two drugs; the most frequent association in those involved the combination of three (n = 86, 5.6%).

**TABLE 7:** ATC third level of drugs most frequently found among serious hemorrhage cases (total of 3,648 drugs).

ATC 3	*n* (%)
Antithrombotic agents	2,537 (70)
Antidepressants	179 (5)
Antiarrhythmics, classes 1 and 3	142 (4)
Beta-lactam antibacterials, penicillins	115 (3)
Antiinflammatory and antirheumatic products, nonsteroids	88 (2)
Lipid modifying agents, plain	62 (2)
Quinolone antibacterials	60 (2)
Macrolides, lincosamides, and streptogramins	50 (1)
Other beta-lactam antibacterials	48 (1)
Corticosteroids for systemic use, plain	46 (1)

Among the 11 cases without antithrombotic agents, 5 cases were found with an antidepressant, including 3 cases with an NSAID (Table 8).

**TABLE 8:** Drug therapeutic/pharmacological subgroup combinations involved in hemorrhage cases without antithrombotic agents, *n* = 11.

ATC 3	*n* (%)
All other therapeutic products + drugs for constipation	1 (9)
Antidepressants + antidepressants	1 (9)
Antidepressants + drugs for peptic ulcer and gastroesophageal	1 (9)
Antidepressants + NSAIDs	3 (27)
Antifungals for systemic use + corticosteroids for systemic use, plain + immunosuppressants + immunosuppressants	1 (2)
Anxiolytics + hormonal contraceptives for systemic use	2 (18)
Antimetabolites + other antineoplastic agents	1 (9)
Bacterial vaccines + bacterial and viral vaccines, combined	1 (9)

## Discussion

ADRs identified as resulting from DDIs constituted only 2.1% of the spontaneous reports recorded in the French Pharmacovigilance Database from 2012 to 2016. The reports of DDI-ADR concerned men and older patients more than the reports of ADR in general.

More than 80% of reported DDI-ADRs were serious and 7% were fatal; this fatality rate was twice higher than that observed overall for all the reported ADRs. The drugs most frequently incriminated in the occurrence of serious DDI-ADRs were antithrombotics (more than a third) and antidepressants (5%); a third of the informed DDIs for those serious DDI-ADRs involved at least three interacting drugs. In terms of clinical consequence, hemorrhage constituted around half of the provoked serious adverse reactions and two-thirds of the fatal ones. The DDIs involved in their occurrence resulted mostly from the association of two antithrombotics or from the association of antithrombotics and antidepressants.

The proportion of spontaneously reported ADRs resulting from a DDI is 2.1% in this study. As compared to the literature where it can range from 5 to 20%, this appears low (Peyriere et al., 2003; Leone et al., 2010; Bénard-Laribière et al., 2015). Our finding could either result from an under coding of interactions and DDI-ADRs in French Pharmacovigilance Centers or from a specific underreporting of these ADRs in France. The latter hypothesis could imply that DDI-ADR are less considered when attributed to the drug than to the prescriber and thus would be less needed to be reported in terms of safety evaluation of the drugs. In the same perspective, it could also be that prescribers would be less prone to report such events for which they would potentially be responsible, a barrier to reporting that has already been well identified in the literature (Mirošević Skvrce et al., 2011). This would however paradoxically be less true for serious DDI-ADR than for nonserious one, as the seriousness rate appears high for DDI-ADRs compared to all reported ADRs. However, in that situation, often implying hospitalization, the reporter could be more likely to be the clinician responsible for the ADR management rather than the prescriber.

This study showed that the majority of serious DDI-ADRs were related to antithrombotic agents (anticoagulant or antiplatelet drugs), consistent with the literature which clearly identifies hemorrhage as a well-known consequence of a serious DDI (Pirmohamed et al., 2004; Leone et al., 2010). Even with its intrinsic limitation of underreporting, this study confirmed, in this perspective, that this issue is still involving the occurrence of a very large number of serious hemorrhagic accidents due to DDI. Additionally, it highlighted on this subject that 30% of the drugs involved in these hemorrhages are not antithrombotic agents and allowed observing that the association of antithrombotic plus antidepressants was responsible for a significant proportion of serious cases of hemorrhage relating to DDIs. This interaction is well known and led to a warning in the French recommendations regarding DDIs (Interactions médicamenteuses, 2017). However, this recommendation nowadays considers the DDI involving this combination to be a Level 1 (over four) in terms of associated risk. The results make a plea for reconsidering this classification. If its extent was not expected, the finding of this DDI is consistent with the literature in which this DDI is highlighted (Schelleman et al., 2011) even its mechanism remains only partly elucidated, at least for its pharmacokinetic aspects (Sansone and Sansone, 2009). Some studies suggested that citalopram is possibly one of the antidepressants with the lowest risk of interaction because it is a weak enzyme inhibitor of CYP P450 (Brøsen and Naranjo, 2001; Sansone and Sansone, 2009). However, in our study, its derivative, escitalopram appeared to constitute the antidepressant most often involved in bleeding events; however, this finding is only related to its wide use in France. Since Selective Serotonin Reuptake Inhibitors (SSRIs) can cause thrombocytopenia and bleeding (by lowering platelet serotonin and inhibiting subsequent serotonin-mediated platelet activation), this may actually be a pharmacodynamic rather than a pharmacokinetic interaction (Laporte et al., 2017). Another interesting result is that fluindione was the drug most often involved in hemorrhage; in 11% of cases for which it was reported, amoxicillin was the other drug suspected. A similar association was found between warfarin and amoxicillin in 15% of cases for which warfarin was reported. If publications exist (Farnier et al., 2016), responsibility for an interaction between these drugs or with the underlying infection is still debated (Zhang et al., 2011).

Although hemorrhage is one of the most expected serious side-effects after a DDI, their overrepresentation in this study suggests that other DDIs could be less easily identifiable by healthcare professionals. For instance, renal failure was the second clinical consequence observed in this study but represented only 8% of cases. This could be attributed to the fact that this condition can find multiple etiologies other than iatrogenic etiology and would thus be less likely to be identified as such when it is the case (Perazella, 2018). The same goes for arrhythmias which only represented 4% of reported DDI-ADRs, although it constituted one of the most expected ADR resulting from DDI in the literature (Dechanont et al., 2014; Holm et al., 2014; Létinier et al., 2019).

The main strength of our study was focusing on actual clinical consequences of DDI and not on potential interactions. Another strength of our work was that 99% of serious cases were reported by healthcare professionals and 100% of cases were evaluated by a pharmacologist. The use of the BNPV is particularly interesting for more in-depth studies of such a complex pharmacological problem as DDI. Finally, our study was not limited to the interactions involving two drugs. In fact, more than a third of the cases found included at least three suspect drugs; this result advocates for the conduct of further evaluations regarding these more complex situations of drug interactions. However, our descriptive study did not allow us to go further in the analysis of multiple interactions. We are working on the development of specific analytical methods that we will present in future work. The main limitation of our study relates to the underreporting inherent in pharmacovigilance databases (Hazell and Shakir, 2006), which limits the representativeness of our results. Our results suggest that this undernotification would be even stronger for DDI-ADR than for ADR in general. Indeed, it is possible that healthcare professionals are reluctant to report ADR-DDI when it is a well-known mechanism. However, we observe a very high proportion of hemorrhages that are well-known DDIs, so it could be that the difficulty of diagnosing less known DDIs explains this undernotification. Finally, our data were only French and we cannot exclude specific national features in our results. Despite the specificity of the French Pharmacovigilance system, the lessons of this study might be transposed to most countries with similar drug use.

## Conclusion

Hemorrhagic accidents are still accounted for as an important part of ADRs resulting from DDIs reported in France and did not only concern antithrombotic agents. Despite being known in theory, these interactions still seem to be present in current practice. In addition, most of the interactions leading to serious cases are not referenced as a high level of severity interactions in the various DDI reference documents. Taken together with the finding that more than one-third of serious DDI-ADRs involved at least three drugs and are more difficult to identify, this highlights the need for tools dedicated to professionals and patients for better management of DDIs and improving the reporting of DDI-ADRs.

## Data Availability

The data analyzed in this study were subject to the following licenses/restrictions: The French Pharmacovigilance Database is reserved for use by competent authorities. Requests to access these datasets should be directed to Mehdi.BENKEBIL@ansm.sante.fr.
